# STUDY OF TOTAL ANTIOXIDANT STATUS AND GLUTATHIONE PEROXIDASE ACTIVITY IN TUNISIAN VITILIGO PATIENTS

**DOI:** 10.4103/0019-5154.48978

**Published:** 2009

**Authors:** Akrem Jalel, Mohamed Hédi Hamdaoui

**Affiliations:** *From Research Unit on the Antioxidant Compounds, Oxidative Stress, Trace Elements and Metabolic Diseases, Ecole Superieure des Sciences et Techniques de Tunis Health, Tunisia*

**Keywords:** *Diabetes*, *dysthyroidism*, *glutathione peroxidase activity*, *total antioxidants status*, *vitiligo*

## Abstract

**Background:**

Vitiligo affects one to two percent of the word population. Its pathogenesis has not been clarified yet. Multiple mechanisms such as autoimmune, neuronal, endocrine and oxidative stress resulting from unbalanced antioxidant defense system have been proposed.

**Aims:**

Our purpose was to study the total antioxidant status and glutathione peroxidase activity in Tunisian vitiligo patients with or without diabetes or dysthyroidism.

**Materials and Methods:**

We studied 60 vitiligo patients and 62 healthy controls. The sex ratio male/female in vitiligo patients was (27/33 = 0.81). Patients with vitiligo were divided into three groups, according to the association with diabetes or dysthyroidism. The total antioxidant status (TAS), glutathione peroxidase activity (GPX activity) was evaluated by adaptable methods using Kits.

**Results and Conclusion:**

The generalized vitiligo was the most frequent type (35 patients versus 25 of focal ones). All patients having vitiligo showed low levels of TAS: 0.85 ± 0.7 and low GPX activity: 45 ± 0.6, as compared to the control group: 1.40 ± 0.12 mmol/L; 49 ± 1.8 U/L, (p < 0.01), for TAS and GPX, respectively. The association of low TAS and GPX activities was more pronounced in diabetic vitiligo patients than in dysthyroid vitiligo patients. This study demonstrated that antioxidant processes depletion (low TAS and low GPX activity) is clearly involved with vitiligo in Tunisian patients, regardless of the association of the disease with diabetes or dysthyroidism.

## Introduction

Mammalian pigmentation results from the synthesis and accumulation of photoprotective epidermal melanins. Melanins are formed from the amino acid precursor L-tyrosine, within specialized cells, the melanocytes. The melanogenic pathway involves the formation and polymerization of reactive oquinones.[1] To date, systemic and epidermal oxidative stress via H_2_O_2_ in the depigmentation disorder vitiligo has been suggested to be the initial pathogenesis event in melanocyte degeneration in the epidermis of patients with active disease.[2–4] Detailed *in vitro* and *in vivo* studies on various metabolic mechanisms have provided a new understanding of the physiological and physio-pathological events in this disease process, despite the fact that the precise step in the cascade for initiation of this peculiar disorder still remains unknown.[5,6] Vitiligo has been also reported in association with several endocrinopathies and other disorders of autoimmune nature such as dysthyroidism and diabetes. Our purpose was to examine the total antioxidant status and glutathione peroxidase activity in Tunisian vitiligo patients with or without associated metabolic diseases.

## Materials and Methods

### Patients

Sixty vitiligo patients and sixty two healthy volunteers aged from 11 to 67 years attending in Medenine Hospital (Medenine is a town in the south of Tunisia) participated in the present study. Local medical authority has approved the study and informed consent has been obtained from each subject. The total vitiligo patients (TVP, n = 60) were subdivided into three groups: vitiligo patients without associated pathologies (VP, n = 25), diabetic vitiligo patients (DVP, n = 20) and dysthyroid vitiligo patients (ThVP, n = 15). All subjects were interviewed according to a standard questionnaire for details of their age, demographic, clinical, anthropometrics (BMI, kg/m^2^), family vitiligo, diabetes and dysthyroidism history, smoking habits, and their habits diet information, in particular type of foods, meals, desserts, snacks and drinks consumed including coffee and tea, site of onset, duration, and past treatment were taken. A complete clinical examination was done, and the site and pattern of the lesions were noted. The evolution of the disease, as evidenced by the appearance of new lesions and increase in the size of existing lesions over the past three months was also completed. Screening was done for autoimmune and endocrine disorders by history and clinical examination. These disorders included thyroid disease and diabetes mellitus. Investigations including thyroid hormones (TSH, T3), plasma glucose are determined for each patient.

### Blood sampling

Fasting blood was collected and was subdivided in aliquots. One of them was used to analyze basal clinical parameters. Another was used to determine the whole blood GPX Activity (GPX). Remain of blood was centrifuged at 3,000 rpm for 10 min, and then the plasma was removed and frozen for analyses of total antioxidant status (TAS).

## Analysis of samples

### Analyses of basal clinical parameters

Fasting blood glucose (FBG) was measured by routine Auto analyzer methods (Synchron CX 7, Beckman), using dedicated kits. T3 was measured by resin fixation and TSH by radioimmunology.[7]

### Analyses of total antioxidant status, whole blood GPX activity

The TAS was determined in heparinized plasma samples by the method of Miller *et al*.[8] The whole blood GPX activity was determined by the method of Paglia and Valentine. Kits for analysis of TAS and GPX activities were purchased from Randox (United Kingdom).

### Statistical analysis

Statistical analyses were carried out using ANOVA followed by Student's t-test for normally distributed data or by the Mann-Whitney U-test for non-normally distributed data. Associations between variables were examined using Spearman's rank correlation coefficient. All data are presented as mean ± SEM. Differences were considered significant at p < 0.05.

## Results

The vitiligo characteristics (type, age at onset and sex ratio) of Tunisian patients are given in [Table 1]. The sex ratio was (27/33 = 0.81) and the generalized vitiligo was the most frequent type (35 patients versus 25 of focal ones). Anthropometric characteristics of different groups (age, weight and BMI) are indicated in [Tables 2 and 3]. They revealed overweight as indicated by their BMI ≥27kg/m^2^. Fasting blood glucose was significantly higher in diabetics than in controls (p < 0.01). In dysthyroid vitiligo patients, the TSH significantly increased, while the T3 showed to be decreased. Values of TAS and GPX are given in [Table 3]. The total antioxidant defense system decreased in diabetics groups. The values of TAS were normal in the control group as compared to the reference values (between 1.3 and 1.7 mmol) given by RANDOX. However, values of the TAS were significantly lower the control group. The GPX activities decreased in all group of vitiligo by about 40% or more, in particular in DVP group (45%).

**Table 1:** Vitiligo characteristics of Tunisian patients

Characteristics	Vitiligo (n=60)	Healthy controls (n=62)
Number of male/female	27/33	30/32
Early onset/late onset	16/44	
Clinical type(generalized/focal)	35/25	
With/without other autoimmune diseases	26/34	

**Table 2:** Anthropometric and clinical characteristic of subjects

Parameters	Control group (n = 62)	TVP (n = 60)	VP (n = 25)	DVP (n = 20)	ThVP (n = 15)
Age (years)	42 ± 6	43 ± 9	30 ± 8	54 ± 10	45 ± 11
Weight (kg)	65 ± 9	75 ± 12	71 ± 13	79 ± 15	77 ± 15
BMI (kg/m^2^)	25 ± 2	27 ± 3	27 ± 4	28 ± 5	26 ± 5
Plasma glucose, mmol/L	5.5 ± 0.1	5.6 ± 0.7	5.2 ± 0.1	7.8 ± 0.1**	3.7 ± 0.1
TSH mU/L	0.43 ± 0.4	-	-	-	0.76 ± 0.3***
T3 nmol/l	1.9 ± 0.8	-	-	-	0.43 ± 0.6***

** Significantly different from the control group: p < 0.001,

*** significantly different from the control group: p < 0.01

**Table 3:** Total antioxidant status and glutathione peroxidase activity

Parameters	C (n = 62)	TNV (n = 60)	VP (n = 25)	DVP (n = 20)	TVP (n = 15)
TAS, mmol/L	1.4 ± 0.12	85 ± 02***	0.95 ± 0.3**	0.82 ± 0.2***	0.8 ± 0.03***
GPX, U/L	49 ± 1.8	42 ± 3**	45 ± 1**	41 ± 2***	43 ± 3***

*** p < 0.001;

** p < 0.01

## Discussion

One of the major hypotheses in the pathogenesis of vitiligo is the oxidative stress induced by reactive oxygen species (ROS) which plays a major role in the production of free radicals. In spite of its key role in the regulation of proliferation and differentiation of many cell types, ROS usually causes several damages of different cell types. Epidermal melanocytes are concerned and the potential regulatory roles of ROS might be particularly important, as ROS are frequently generated in epidermal cells following UV irradiation (normal tanning stimulus but also the main etiological factor for skin cancer). In addition to this excessive production of ROS, there are other pathways conducting to oxidative stress like defective recycling of tetrahydrobiopterin which has been reported in vitiligo epidermis.[5,9] The alteration in the antioxidant pattern, with a significant reduction of GPX and catalase activities has been demonstrated in both lesional and non lesional epidermis of patients.[10,11] The antioxidant imbalance has been confirmed also in peripheral blood mononuclear cells of active vitiligo and was correlated to the increased intracellular production of reactive oxygen species and appeared to be a consequence of mitochondrial impairment.[9,12] These findings support the concept of a possible systemic oxidative stress in vitiligo. The generation of ROS and/or the resulting increase in lipid peroxidation products have been proposed as etiological factors for depigmentary natural processes such as hair graying[13] and several pathological conditions, like vitiligo.[8,14] Moreover, imbalances of the normal antioxidant mechanisms are common in human melanoma cells[15] and the autocytotoxic hypothesis suggests that melanocyte impairment could be related initially to an increased oxidative stress,[16,17] with a consequent induction of H_2_O_2_ accumulation in the epidermis of patients with active disease,[18] lower levels of GPX and catalase were demonstrated in the epidermis of both lesional and non lesional skin of vitiligo patients[19,20] and during active phases of disease, an imbalance of antioxidants was found in both the epidermis[21] and peripheral blood mononuclear cells (PBMC), correlated to an increased intracellular ROS production.[22,23] These data, in accord with our findings of decreased levels of GPX and TAS, suggest that the entire epidermis, and even PBMC, may be involved in vitiligo etiology. H_2_O_2_ and other reactive oxygen species are key regulators of many intracellular pathways, within mammalian skin. The polymerization reactions involving quinonic melanogenic intermediates are spontaneous, and lead to H_2_O_2_ production. In support of this view, several oxidative reactions of melanin precursors are inhibited by catalase.[1] In active vitiligo an increased oxidative stress of the entire epidermal compartment has been demonstrated.[16,21] Recently, the activity of vitiligo has been associated with a systemic oxidative stress, evaluated by assessing the intracellular generation of ROS and the antioxidant pattern in PBMC.[24] In particular, TAS, GPX, glutathione and Vitamin E levels were decreased, and this imbalance of antioxidants was associated with hyperproduction of ROS.[25–28] These aspects of oxidative stress are related to cytoplasmic events. In the B16 mouse melanoma model, this inhibition is not related to a severe cellular damage, as treatment conditions with a strong inhibitory action, are adequately withstood by the cells.

## Conclusion

The results of the present investigation provide the first evidence for an important role of oxidative stress in the physiopathology of vitiligo.
